# The Relationship between Maternal Personality Disorder and Early Birth Outcomes: A Systematic Review and Meta-Analysis

**DOI:** 10.3390/ijerph17165778

**Published:** 2020-08-10

**Authors:** Claire A. Marshall, Julie Jomeen, Chao Huang, Colin R. Martin

**Affiliations:** 1 Perinatal Mental Health Liaison Team, Humber Teaching NHS Foundation Trust, Hull HU2 8TD, UK; Claire.Marshall@hull.ac.uk; 2 School of Health and Human Sciences, Southern Cross University, Lismore, NSW 2480, Australia; julie.jomeen@scu.edu.au; 3 Hull York Medical School, University of Hull, Hull HU6 7RX, UK; C.Huang@hull.ac.uk; 4 Institute for Clinical and Applied Health Research (ICAHR), University of Hull, Hull HU6 7RX, UK

**Keywords:** personality disorder, preterm birth, low birth weight, APGAR

## Abstract

(1) Background: Women with personality disorder are at risk of social and emotional problems which impact deleteriously on everyday functioning. Moreover, a personality disorder diagnosis has been established to have an adverse impact upon pregnancy outcomes and child health. Understanding this impact is critical to improving both maternal and child outcomes. This systematic review and meta-analysis will evaluate the contemporary evidence regarding these relationships. (2) Methods: Prospero and Cochrane were searched for any systematic reviews already completed on this topic. Academic Search Premier, CINAHL Complete, MEDLINE, PsycARTICLES, PsycINFO via the EBSCO host, and the Web of Science Core Collection were searched to include research articles published between 1980 and 2019. A total of 158 records were identified; 105 records were screened by reviewing the abstract; 99 records were excluded; 6 full text articles were assessed for eligibility; 5 records were included in the review. (3) Results: All the included studies reported on preterm birth. The meta-analysis indicates significant risk of preterm birth in women with personality disorder (overall odds ratio (OR) 2.62; CI 2.24–3.06; *p* < 0.01). Three studies reported on low birth weight, with the meta-analysis indicating a raised risk of low birth weight of the babies born to women with personality disorder (overall OR 2.00 CI 1.12–3.57 (*p* = 0.02)). Three studies reported on appearance, pulse, grimace, activity, and respiration (APGAR) score, with the meta-analysis of OR’s indicating a risk of low APGAR score in women with personality disorder (overall OR 2.31; CI 1.17–4.55; *p* = 0.02). (4) Conclusions: The infants of women with personality disorder are at elevated risk of preterm birth, low birth weight and low APGAR score.

## 1. Introduction

There has been much interest in the relationship between alcohol misuse use during pregnancy and deleterious neonatal outcomes, particularly in terms of links to associated developmental disorders, such as foetal alcohol syndrome [1,2,3]. Comparatively under-researched within the pregnancy and childbirth context is the relationship between personality disorder and impoverished neonatal outcomes, a perhaps surprising observation given the relationships observed between alcohol misuse during pregnancy, personality disorder and personality traits [4,5].

Personality disorder is defined in the Diagnostic and Statistical Manual of Mental Disorders-5 (DSM-5) [6] as “an enduring pattern of inner experience and behavior that deviates markedly from the expectations of the individual’s culture, is pervasive and inflexible, has an onset in adolescence or early adulthood, is stable over time, and leads to distress or impairment” [6].

A systematic review and meta-analysis established a prevalence rate of personality disorder of 12.5% in the general adult population across Western Countries [7]. A worldwide systematic review concluded that prevalence estimates for psychiatric outpatients in Europe ranged between 40% and 90%, 45% and 51% in the USA and 1.07% in India, and 60% in Pakistan [8]. These inconsistencies highlight issues in accurate epidemiological accounts of personality disorder, it being suggested that both profound methodological issues and tension between diagnostic systems may be explanatory factors [9]. These observations present challenges for the identification, treatment, and outcome optimization of personality disorder. Key concerns in terms of identification relate principally to overlap both within personality disorder clusters and with other disorders, such as the comorbidities described in the literature with particular reference to borderline personality disorder, bipolar disorder, major depressive disorder, anxiety, posttraumatic stress disorder, psychosis, or other psychotic disorders [10,11].

There has been ongoing debate in the academic literature highlighting problems associated with the International Classification of Disease, 9th revision (ICD 9) [12] and the Diagnostic and Statistical Manual of Mental Disorders, 5th edition (DSM-5) [6] categorical approaches to personality disorder diagnosis, including arbitrary thresholds, diagnostic overlap and lack of use in clinical practice [13]. The diagnosis of personality disorder requires specialized training and can be complex in comparison to the diagnosis of other mental disorders. Therefore, in the soon to be published International Classification of Disease, 11th revision (ICD 11), it is proposed that personality disorders will be classified by using a dimension structure of severity: mild, moderate or severe [14]. There are concurrent debates suggesting that borderline personality disorder is included as a distinct entity in the ICD 11 version, albeit an enhanced version of the current definition of post-traumatic stress disorder (PTSD), recently described as “complex PTSD” [15]. Complex PTSD refers to the degree of precipitating traumatic events that may be single, prolonged or repeated but with effects that are severe and devastating, leading to profound psychological disturbance [16].

In clinical practice, there has been criticism of the diagnosis of personality disorder, due to concerns about the diagnosis being used as a method for excluding individuals from treatment [17,18]. The diagnosis may be applied to people who are perceived as too difficult to treat or untreatable, or to mark out individuals to receive specialist services rather than be treated within generic mental health services, despite the diagnosis being a common yet challenging long term condition [19]. Stigma associated with the personality disorder diagnosis is a concern [20] and there has been a growing movement to acknowledge the relevance of childhood trauma and adverse childhood experience with identity disturbance and interpersonal difficulties, when considering the diagnosis [21].

The difficulties associated with personality disorder can pass through the generations of a family, possibly occurring through epigenetic programming [22]. An integrative model has been proposed to describe the multifactorial aetiology of the condition, including a genetic vulnerability to environmental factors [23] which may be sustained by diverse neurobiological factors [24]. It has been observed that women with personality disorder are at a greater risk of unplanned pregnancy, sexually transmitted disease, and lack of social support [25]. Rates of domestic violence are higher in this population [26], and studies have shown that violence in pregnancy can have an effect on neonatal outcomes, including preterm birth [27]. Women with personality disorder may have difficulty in adjusting to motherhood and there is a risk of emotional and behavioral problems impacting on their offspring through the generations of a family [28].

Women with personality disorder have recently been described as “high risk” caregivers in order to explain the complex interaction between their psychological and interpersonal functioning, and their relationship and social difficulties [29]. There are higher rates of risk-taking behavior that impact the fetus, such as drug and alcohol misuse [30], increased rates of smoking in pregnancy [25], and poor nutrition. Troubled relationships, including domestic violence, may also lead to negative birth outcomes for the baby in relation to preterm birth, lower appearance, pulse, grimace, activity, and respiration (APGAR) scores, and lower birth weight [31].

It is important to understand the morbidity and mortality that a diagnosis of personality disorder might have and the associated treatment implications across the life course [32], including the neonatal outcomes for a woman with personality disorder who is pregnant. These key risk factors in the lives of women with personality disorder can significantly complicate the pregnancy and birth process and have implications for the mother and baby, wider family, and society in the short and longer term [29].

There has been a focus in the research, policy, and in perinatal mental health service design internationally on perinatal depression, anxiety, and bipolar disorder or other psychotic illnesses [33,34]. In many areas, a lack of policy and treatment guidance for services for pregnant women with personality disorder has led to an exclusion of women with personality disorder from specialist perinatal mental health services which brings service strategy and planning issues, and potentially leaves the most vulnerable without a service and without clear treatment plans [29].

A number of studies have examined the early outcomes for the children of women who have diagnoses of schizophrenia, mood disorder and depression [29,35]. Women with bipolar disorder, for example, are at greater risk of having a preterm and low birth weight infant [27,36,37]. Yet, few studies have attempted to examine the outcomes for the babies of women with personality disorders. Mechanisms underlying the risk of adverse outcomes for women with mental illness are not clear; however, some studies have highlighted the potential genetic risks crossing mental illness groups due to a number of susceptibility genes being shared across disorders, such as borderline personality disorder, bipolar disorder, major depression, and schizophrenia [38].

Other studies have suggested that the increased poor pregnancy outcomes within women experiencing mental illness are linked to the clustering of adverse maternal risk factors that are associated with poor birth outcomes, including hazardous lifestyle factors, smoking, drug and alcohol misuse, poor diet, and lack of exercise [39]. Mediating factors for these risk issues, such as referral to smoking cessation courses [40], or health and lifestyle advice, are particularly challenging for women with personality disorder as these women often have a low level of engagement with maternity and antenatal care [41].

Key physiological factors in the new born which may be influenced by maternal personality disorder include (i) pre-term birth, (ii) depressed APGAR score, and (iii) low birth weight. These factors are selected as outcomes in this systematic review due to their routine use globally, and the well documented impact on the development and functioning of the infant across the life course including the increased risk of developmental, neurological, respiratory and behavioral problems.

The characteristics of these indices will be briefly discussed.

### 1.1. Preterm Birth

Preterm birth describes infants that are born at less than 37 weeks’ gestational age [42,43]. Preterm births account for around 5% of births in high income countries and 25% in low-middle income countries [44]. 

Complications from preterm birth are a primary cause of neonatal death worldwide and can result in lifelong effects on functioning such as increased risk of cerebral palsy, learning disability and visual disorders, and an increased risk of chronic disease in adulthood. There is a significant impact on the individual and family throughout the life course [45]. The economic cost of pre-term birth has been described as 2.9 billion pounds annually in the UK [46], due to the high cost of intensive care, and ongoing health, social and educational needs.

### 1.2. APGAR

The APGAR (appearance, pulse, grimace, activity, and respiration) score is used to assess a new born’s health using five dimensions, and despite being developed over 60 years ago remains a valid assessment of the baby’s wellbeing when assessed at 1 min and 5 min post birth. A low APGAR score correlates with neonatal mortality in large populations [47]. Scores above 7 are considered normal, scores between 4 and 6 low and below 3 are considered critically low. The APGAR score is universally used to assess newborn’s health, with studies finding that low APGAR score at 5 min is associated with substantially increased risks of neonatal and infant mortality, both in preterm and term infants [48]. It is considered a standardised, effective, and convenient tool for neonatal assessment, supported by its global use [49,50,51]. The prevalence of APGAR scores <7 internationally ranges between 0.3% and 2.4% [52].

### 1.3. Low Birth Weight

Low birth weight (LBW) is defined by the World Health Organisation (WHO) as less than 2500 g (5.5lb) [53]. Like preterm birth, low birth weight is a significant predictor of prenatal mortality or morbidity, and increases the risk of long-term health problems across the lifespan [54], with babies of LBW being 20 times more likely to die earlier than their heavier counterparts [53]. Conservative estimates of LBW prevalence made by UNICEF and the WHO in 2004 suggested that at least 16% of births globally were LBW, with around 96% of these in low to middle income Countries [53]. LBW is not a proxy for a unidimensional measure of maternal or infant health, rather it is an indicator (particularly in resource limited settings), for multifactorial health, economic, and social factors, which have long term implications reflecting an ongoing risk of negative health and social outcomes [54,55].

## 2. Objectives

- To conduct a systematic review to identify the impact of personality disorder of the mother on risk of preterm birth <37 weeks gestation, birth weight <2500 g, and APGAR score <7;
- To assess the quality of the research literature identified through the systematic search strategy;
- To conduct a meta-analysis to synthesis the data on preterm birth, birth weights, and APGAR score where data is available.

## 3. Methods

### 3.1. Eligibility Criteria

1. Studies which include mothers with a diagnosis of personality disorder or identified through the study as meeting diagnostic criteria of a personality disorder of any type.
2. Studies reporting neonatal outcomes (specifically preterm birth <37 weeks gestation, APGAR scores <7, and low birth weight <2500 g).

### 3.2. Exclusion Criteria

1. Studies are excluded if they are review articles, expert opinion commentaries, or single case report.
2. Studies are excluded if they are not written in English language.
3. Diagnostic criteria for personality disorder were changed significantly with the introduction of the Diagnostic and Statistical Manual version III in 1980; therefore, studies published before this will not be included.

### 3.3. Information Sources

The Cochrane database and Prospero were searched for systematic reviews relating to women with personality disorder and early birth outcomes. The search strategies were developed using medical subject headings (MeSH) and text words related to the population and outcome of interest (personality disorder and preterm birth, low birth weight, and APGAR score). Academic Search Premier, CINAHL Complete, MEDLINE, PsycARTICLES, PsycINFO via the EBSCO host, and the Web of Science Core Collection.

The key terms used within the search were: premature deliver, preterm birth, intrauterine growth restriction, small for gestational age, APGAR, birth weight, birth size, neonatal intensive care unit (NICU), paediatric intensive care, preterm labour, preterm infant, premature baby, and personality disorder. The search was undertaken in October 2018, and excluded articles published prior to 1980.

### 3.4. Study Records

The title and abstract of papers identified in the initial search were screened to determine whether full text of the article should be reviewed. If a title appeared relevant and the abstract was available then the full text was requested. A flow chart detailing the number of studies identified in the search strategy was used, and details the study selection process.

### 3.5. Management

A data extraction form was created based on Preferred Reporting Items for Systematic Reviews and Meta-Analyses (PRISMA-P) [56]. Data was extracted and summarized in table format, and included name of authors, year of publication, name of country in which the study was conducted, study design, sample size, method used to identify personality disorder, preterm birth, APGAR, and low birth weight > 2500 g.

### 3.6. Selection Process

Studies have been screened for adherence to inclusion/exclusion criteria. Screening for study selection and inclusion was completed by the lead author.

### 3.7. Critical Appraisal of Studies

A quality appraisal of each study has been undertaken, assessing each study against the Critical Appraisal Skills Programme (CASP) [57].

### 3.8. Meta-Analysis

Meta-analysis was undertaken using a random effects meta-analysis model. Pooled estimate of the odds ratio (OR) and associated 95% confidence interval (CI) was reported. The statistical significance was set at *p* < 0.05. Heterogeneity for studies was presented by I^2^ All analyses were conducted with Review Manager (RevMan) 4.3 (Review Manager [Computer program]. Copenhagen: The Nordic Cochrane Centre, The Cochrane Collaboration). 

## 4. Results

There were no systematic reviews identified through the search of the Cochrane database and Prospero. Using the search strategy previously described, 158 records were identified, with 105 records once duplicates were removed. There were no records identified from other sources such as reference lists. During the screening process, 99 records were excluded. Of these records, 98 were excluded as they did not report on both the population of interest (women with personality disorder) and neonatal outcomes.

There were two studies with missing data or data not fully reported in relation to the population of interest (women with personality disorder), and the outcomes of preterm birth, APGAR, and low birth weight <2500 g. The lead author of each paper was contacted by email to request data [26,58]. One author [26] responded and provided the requested data.

**Figure 1:**
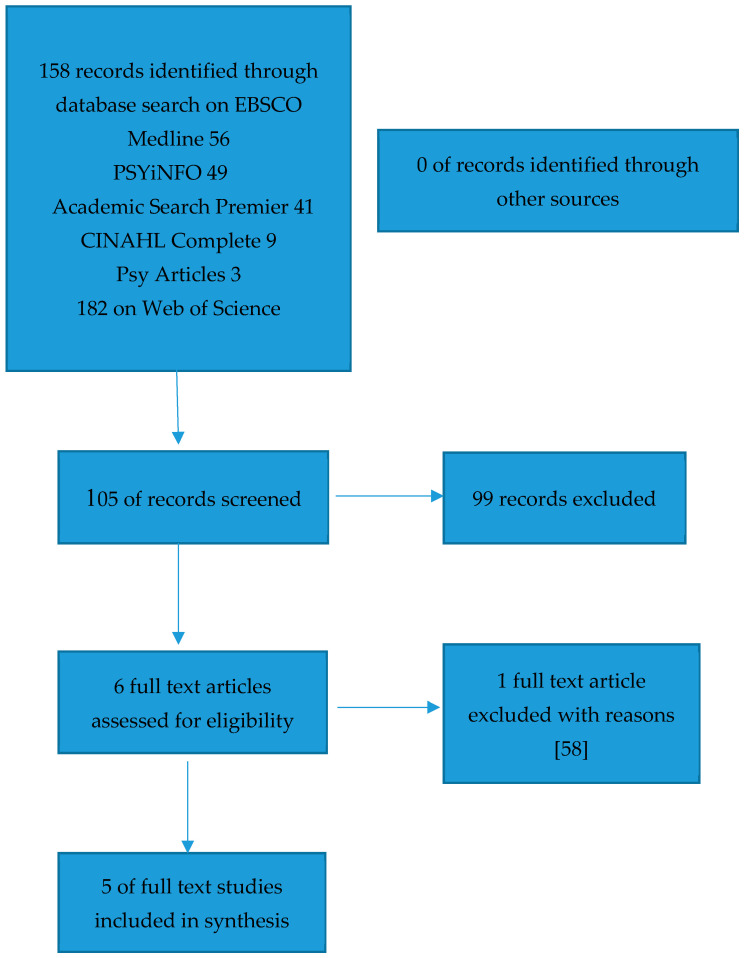
Flowchart detailing the inclusion and exclusion of studies.

Without additional information it was not possible to include one [58] study in this systematic review as no specific outcomes in relation to women with personality disorder and preterm birth, APGAR, or birth weight <2500 g. The study retrieval process is summarized in Figure 1.

### 4.1. Study Summary

Five studies were included in this systematic review and meta-analysis [26,41,59,60,61]. From the included studies, 1114 women were identified as having a personality disorder and 8,527,780 women formed the control group. This information is shown in Table 1.

### 4.2. Population of Study/Identifying the Population

Two of the included studies [41,59] focused on women with borderline personality disorder. One study included women with antisocial personality disorder [26], and a further two studies did not specify the type of personality disorder of the women in the study [60,61]. Three studies [26,60,61] included women with a number of different mental illnesses and then looked at outcomes against a number of different diagnosis of mental illness. One of the studies considered the specific and combined role of domestic violence and mental illness on new-born health [26].

There are differences across the studies regarding the methods employed to identify women with personality disorder, and this may be reflected in the wide range of prevalence rates of personality disorder across the included studies. Within the included studies, the rates of women with personality disorder appear low in relation to the previously stated prevalence rates in the psychiatric community population [7,8]. One study [41] included a population of women within a specialist perinatal mental health service, despite this, the prevalence of women with personality disorder remains low in this study. Table 1, details the included studies.

### 4.3. Prevalence Rates of Personality Disorder

There was significant variability of the prevalence of personality disorder within the studies, specifically, 0.012% [59], 0.00764% [60], 0.3% [41], 0.857% [61], and 2.193% [26]. The overall prevalence rate of personality disorder (combining the five studies) is 0.013% (95% CI: 0.012–0.014%). This prevalence rate is low compared to the prevalence rates reported in other studies [7]. The prevalence rate in this systematic review/meta-analysis is largely driven by the very low prevalence rate within one study [59].

### 4.4. Diagnostic Issues and Comorbidity

Four of the studies included ICD criteria and one study used DSM diagnostic coding to identify the population of interest. There has been significant debate about the difficulties with the diagnosis of personality disorder in the theoretical and research literature, and in clinical practice. Across the study there is variability regarding the identification of women with personality disorder. Two studies [59,60] used ICD 9 diagnosis codes taken from hospital records to identify the participants. This is due to the time frame in which data was collected, as ICD 10 was implemented in 2015 following the data collection period of these studies. One study [60] used ICD 10 codes taken from hospital records. One study used patient interviews by trained psychologists to determine potential ICD 10 classification code using the Mini International Neuropsychiatric Interview [26]. The patient population in one particular study [41] appears to be different from the other studies due to it taking place within a specialist perinatal mental health service. The participants had received a diagnosis of borderline personality disorder by the psychiatrist or psychologist within the service, utilizing the DSM IV classification system. This diagnosis was confirmed by the lead researcher ensuring that all participants met a minimum of five of the nine diagnostic criteria for borderline personality disorder.

Personality disorder is associated with high levels of comorbidity, for example 85% of patients with borderline personality disorder have at least one more diagnosis on Axis 1 of the DSM-IV [62] and 74% have another type of personality disorder [10].

There are difficulties in identifying the correct sample in studies of personality disorder, due to the significant level of diagnostic overlap. In particular, bipolar disorder and borderline personality disorder, due to affective instability being a feature of each disorder [63]. This appears to be an issue across all of the included studies.

### 4.5. Baseline Characteristics of the Participants

The literature has identified a number of variables that are likely to be implicated in preterm birth and newborn health. 1. Smoking [64] or substance misuse [65,66]. 2. Ethnicity [67]. 3. Socioeconomic status [68]. 4. Previous preterm birth [69]. 5. Selective Serotonin Reuptake Inhibitor use or another antidepressant use [70]. 6. Obstetric/medical complication [71].

There is variation in the reporting of the baseline characteristics of the participants across the studies. While two of the studies specifically focus on women with mental illness, three of the studies [26,60,61] include women across a number of classifications of mental illness and do not provide data on baseline characteristics stratified by type of mental illness. Age of the mother may play a significant role in preterm birth, and whilst all of the studies collected data relating to the age of participants, only two studies provided details that related to the specific population of patients with personality disorder [41,59], with one study only providing age data relating to the population of patients with personality disorder and not control group [41]. Where data was available [59], women with personality disorder tended to be younger in age than the women in the control group. Only two of the studies [59,60] took note of race/ethnicity of the participants. This is of concern, as there is significant evidence, that race/ethnic group of the mother is associated with poorer birth outcomes, particularly, Black-African women [59]. All of the studies took account of smoking status/tobacco use, and alcohol drug misuse although only one study [59] reported clearly on this for women with personality disorder, and in this study women with personality disorder tended to use alcohol, tobacco, and drugs more commonly than their counterparts in the control group.

### 4.6. Country of Origin of the Studies

Table 1 indicates the geographical location on where the studies took place. Two studies took place in America [59,60] the remaining studies took place in Australia [41], Brazil [26], and Japan [61]. How women are managed in pregnancy may vary due to national and regional protocol and therefore protocols may impact on gestation of pregnancy at delivery. There may be social or cultural influences which impact on policy related to birth such as attitude toward instrumental or surgical interventions in childbirth.

All the included studies took place in middle to high income countries, meaning good access to facilities and equipment in order for accurate measurement during pregnancy of fetal age and weight at birth. However, although measurement techniques and equipment may differ across countries—the outcomes included in this review, preterm birth, APGAR score and low birth weight—are internationally comparative neonatal health indicators which make it possible to assess differences in practices and outcomes.

## 5. Meta-Analysis

### 5.1. Preterm Birth < 37 Weeks Gestation

All five of the included studies reported on preterm birth <37 weeks.

Across the studies there was a range of risk of preterm birth ranging between OR 2.43 (CI 1.16–5.09) [41], OR 2.50 (CI 2.11–2.95) [59], OR 4.40 (CI 1.12–17.31) [61], OR 4.36 (CI 1.37–13.85) [26], and OR 4.85 (CI 2.60–9.04) [60].

**Figure 2:**
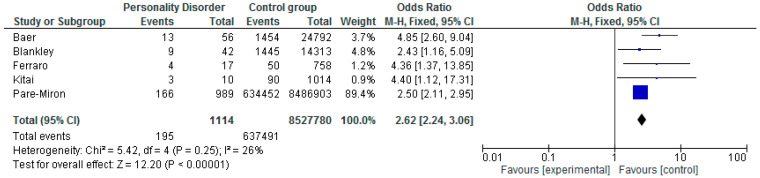
Meta-analysis of preterm birth and personality disorder.

The meta-analysis of OR’s (Figure 2.) indicated significant risk of preterm birth in women with personality disorder (overall OR 2.62; CI 2.24–3.06; *p* < 0.01). The heterogeneity between these studies was low/moderate (I^2^ = 26%).

Within the forest plot, the size of the square corresponds to the weight of each study in the meta-analysis, with larger squares given to studies with larger sample sizes or data. The 95% confidence interval is represented by the horizontal line except for the summary statistic, which is shown by the diamond, the length of which represents the confidence interval.

### 5.2. Low Birth Weight < 2500 g

**Figure 3:**
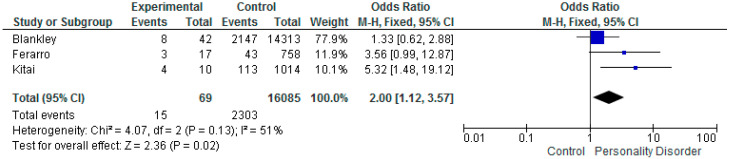
Meta-analysis of studies and low birth weight.

Three of the included studies reports on low birth weight, with the results ranging from OR 1.33 (CI 0.62–2.88) [41], OR 3.56 (CI 0.99–12.87) [26], and OR 5.32 (CI 1.48–19.12) [61]. The meta-analysis of OR’s (Figure 3.) indicated a raised risk of low birth weight of the babies born to women with personality disorder. Overall OR 2.00 CI 1.12–3.57 (*p* = 0.02). The heterogeneity between these studies was (I^2^ = 51%).

### 5.3. APGAR Score Less Than 7

Three of the studies report on APGAR score, OR 19.36: (CI 0.87–426.67) [61], OR 2.17 (CI 1.06–4.41) [41], and OR 3.31 (CI 0.18–61.04) [26], although there were small numbers of participants in the personality disorder group and a small/zero number of events.

**Figure 4:**
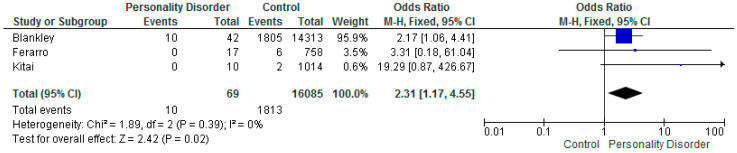
Meta-analysis of studies and APGAR.

The meta-analysis of OR’s (Figure 4.) indicated a risk of low APGAR score in women with personality disorder (overall OR 2.31; CI 1.17–4.55; *p* = 0.02). The heterogeneity for these three studies was low (I^2^ = 0%).

## 6. Discussion

Women with personality disorders experience significant vulnerability and risk factors throughout their life course. The maternal period is no exception and the risks posed to their infants at birth are outlined in this systematic review.

Interpreting the evidence proves challenging for a number of reasons. The diagnostic issues and comorbidity of the disorder can lead to a lack of clarity and consensus, in both the research and clinical arena [19]. The ICD and DSM are classification systems that have evolved over time. The International Classification of Mental Disorders is the most widely used system of medical classification throughout the world [72], so it is unsurprising that it is this classification system that is has been most frequently used to identify the population within the included studies due to its ease of use by non-mental health clinicians, and those working in medical settings and in low and middle income countries. The use of the DSM-IV in one study is of interest as this study was unique from the other studies in that the population of women with personality disorder were taken from an outpatient sample of women under the care of a perinatal mental health team. The DSM and ICD classification can be comparable with good convergence for identifying mental disorders [73]. Considering that the other studies used databases to identify their population it is worth noting that ICD classification is required for each hospital diagnostic record in the US, the DSM is not used. There are further problems within each classification system that relate to poor inter-diagnostic reliability, whereby clinicians do not consistently apply the prescribed structural system in assigning routine clinical diagnoses [74]. Whether the ICD 11 and will bring further clarity is yet to be established.

There are issues with potential confounding variables across the studies, due to the differences in participant inclusion by diagnostic code, which also appears to have influenced the low prevalence of personality diagnosis within the studies. The baseline characteristics of the participants included in the studies, in particular age, ethnicity and substance/alcohol may also bring bias to the overall results of this systematic review/meta-analysis; however, this is difficult to determine as only one study adequately reported the baseline characteristics of the participants with personality disorder and control group.

Women with personality disorder are likely to have lifestyle and social factors that impact on their maternal wellbeing, and in turn impact on the development and wellbeing of their baby at birth [26], and impact upon the mother’s ability to parent. The current literature also appears to indicate that children of mothers with a diagnosis of borderline personality disorder are at greater risk of developing the condition themselves, with theories relating to epigenetics, neurobiological processes, and parental interactions being major risk factors [75].

Research has confirmed an association between obstetric complications and later development of mental disorder; in particular schizophrenia [76,77]. There appears to be some etiologic mechanism contributing to the development of mental illness and the generational and cyclical component of obstetric complication down the maternal line.

When compared to mothers with other types of mental illness, women with personality disorder are likely to be at greater need for support and assistance [78]. This systematic review concludes that women with personality disorder have a raised risk of preterm birth overall OR 2.62; CI 2.24–3.06; *p* < 0.01 and low APGAR score OR 2.31; CI 1.17–4.55 (*p* = 0.02). The meta-analysis found an increased risk of low birth weight OR 2.00 CI 1.12–3.57 (*p* = 0.02).

Comparatively, systematic reviews of women with depression and risk of preterm birth conclude a Risk Ratio (RR) of 1.13 (CI 1.06–1.21) and low birth weight RR 1.18 (1.07–1.30) [79]. In the population of women with Bipolar disorder, women had an increased risk of preterm birth OR 1.83 CI 1.64–2.06) with no increased risk of having a new born that was small for gestational age OR 1.07 CI 0.96–1.21) [80]. These findings indicate that the early infant outcomes for women with personality disorder may be worse than for women with other forms of mental illness.

There is the potential for early identification of women with personality disorder, where targeted health interventions and multidisciplinary management can be implemented in order to reduce poor outcomes for the baby/child and woman. This early identification and support also have the potential to enable the prevention of maladaptive development trajectories within the mother infant relationship [75,78].

The development of robust multidisciplinary care pathways for pregnant women with personality disorder, involving specialist perinatal mental health services that are integrated into maternity and obstetric care, would seem to be a sensible conclusion. Yet, to date, despite significant investment and expansion in perinatal mental health services particularly in the UK with the work of the Five Year Forward View [81], there has been a lack of acknowledgement and inclusion of this particular group of women and their families.

## 7. Limitations

- Both prospective and retrospective study design have been used across the included studies, which may impact on the overall outcome of the systematic review/meta-analysis.
- Only one study [59] provided detailed information regarding baseline characteristics of the study participants with personality disorder, including the control group.
- Different diagnostic classification systems were employed in the studies which has brought variability to the methods used across the studies.
- There were lower than expected prevalence rates of personality disorder across all the included studies, this may be due to under reporting or misclassification. across all studies.
- There were significant differences in the sample sizes, most were small and although there was a large overall affect across the meta-analysis this is due to 1 study [59].

## 8. Conclusions

This systematic review and meta-analysis provide a convincing argument that identifies women with personality disorder and their babies as being significantly vulnerable at a critical time of childbearing, as their babies are at risk of poor birth outcomes. The knowledge of this risk amplifies the already known risk that babies born to women experiencing significant psychological problems such as personality disorder may face. Policymakers and health service managers should take note that the impact of personality disorder on birth outcomes is of concern and women within these vulnerable groups, their families and infants should be incorporated into service planning from the viewpoint of perinatal mental health, obstetric and maternity and pediatric care.

## Figures and Tables

**Table 1:** Description of included studies.

Author Publication Year, Country of Study, Reference	Study Description	Patient Numbers/Participant Details *N* (%)			PD Identification	<37 Weeks	APGAR < 7	Low Birth Weight (LBW) < 2500 g
Pare-Miron et al. 2016 US [59]	Retrospective cohort study. All births in study period 2003–2012. Extracting data from the Healthcare Cost and Utilisation project.	Women with PD *N* = 989 Control Group *N* = 8,486,903			Borderline PD code identified on the database. Using ICD 9 diagnostic code to identify women.	PD *N* = 166 CG *N* = 634,452 OR 2.50 (CI 2.11–2.95)	Does not report	Does not report
		Maternal age	Women without PD	Women with PD				
		<18	265,826 (3.13)	21 (2.12)				
		18-25	2,630,926 (31.00)	437 (44.19)				
		25-34	4,338,214 (51.12)	454 (45.90)				
		>35	1,251,937 (14.75)	77 (7.79)				
		Ethnicity	Women without PD	Women with PD				
		White	3,584,672 (42.24)	601 (60.77)				
		Black	942,881 (11.11)	93 (9.40)				
		Hispanic	1,626,628 (19.17)	56 (5.66)				
		Other	726,829 (8.56)	37 (3.74)				
		Alcohol/Substance Use	Women without PD	Women with PD				
		Tobacco	483,895 (5.70)	388 (39.23)				
		Alcohol	8404 (0.10)	38 (3.84)				
		Drugs	234,290 (2.76)	252 (25.48)				
Blankley et al. 2015 Victoria, Australia [41]	Retrospective case review. Patients under the care of a perinatal service in Australia. January 2010 to June 2012.	Women with PD *N* = 42 Control Group *N* = 14,313			Documented evidence in case file that patient met DSM IV diagnostic criteria. Meeting 5 of 9 DSM criteria.	PD *N* = 9 CG *N* =1445 OR 2.43 (CI 1.16–5.09)	PD *N* = 10 CG *N* = 1,805 OR 2.17 (CI 1.06–4.41)	PD *N* = 8 CG *N* = 2,147 OR 1.33 (CI 0.62–2.88)
		Maternal age Age range 15–43 Mean = 27.43 SD 6.22						
		Ethnicity Information not provided for PD Sample						
		Women with PD Substance use 18 (42.8%)						
Kitai et al. 2014 Japan [61]	Retrospective cohort study in Japan. January 2009 to December 2011.	Women with PD *N* = 10 Control Group *N* = 1014			Women diagnosed by psychiatrist using ICD10. Women with personality and behavioral disturbance.	PD *N* = 3 CG *N* = 90 OR 4.40 (CI 1.12–17.31)	PD *N* = 0 CG *N* = 2 OR 19.29 (CI 0.87–426.67)	PD *N* = 4 CG *N* = 113 OR 5.32 (CI 1.48–19.12)
		Maternal age Data not provided						
		Ethnicity Data not provided						
		Alcohol/Drug Use Data not provided						
Ferarro et al. 2017 Sao Paulo, Brazil [26]	Prospective cohort study. Diagnostic interviews to determine whether any had a mental illness. July 2010 to December 2012.	Women with PD *N* = 17 Control Group *N* = 758			ICD 10 coding specifically for antisocial personality disorder. Trained psychologists used the Mini International Neuropsych Interview (MINI).	PD *N* = 4 CG *N* = 50 OR 4.36 (CI 1.37–13.85)	PD *N* = 0 CG *N* = 6 OR 3.31 (CI 0.18–61.04)	PD *N* = 3 CG *N* = 43 OR 3.56 (CI 0.99–12.87)
		Maternal age Data not provided						
		Ethnicity Data not provided						
		Alcohol/Drug Use Data not provided						
Baer et al. 2016 California, US [60]	Retrospective cohort study of births from 2007 to 2011 in the birth cohort registry. Restricted to women with medical insurance.	Women with PD *N* = 56 Control Group *N* = 24,792			Women identified with ICD codes 9th edition on their hospital discharge records.	PD *N* = 13 CG *N* = 1454 OR 4.85 (CI 2.60–9.04)	Does not report	Does not report
		Maternal age Data not provided						
		Ethnicity Data not provided						
		Alcohol/Drug Use Data not provided						

Note: PD = personality disorder, PTB = preterm birth, LBW = low birth weight, APGAR = appearance, pulse, grimace, activity, and respiration, OR = odds ratio, CI = confidence intervals, ICD = international classification of disease, CG = Control Group.

## References

[1] Greenmyer J.R., Popova S., Klug M.G., Burd L. (2020). Fetal alcohol spectrum disorder: A systematic review of the cost of and savings from prevention in the United States and Canada. Addiction.

[2] Popova S., Dozet D., Burd L. (2020). Fetal Alcohol Spectrum Disorder: Can We Change the Future?. Alcohol. Clin. Exp. Res..

[3] Vorgias D., Bernstein B. (2020). Fetal Alcohol Syndrome.

[4] Gentile S. (2015). Pharmacological management of borderline personality disorder in a pregnant woman with a previous history of alcohol addiction: A case report. Clin. Drug Investig..

[5] Magnusson A., Goransson M., Heilig M. (2007). Hazardous alcohol users during pregnancy: Psychiatric health and personality traits. Drug Alcohol. Depend..

[6] American Psychiatric Association (2013). Diagnostic and Statistical Manual of Mental Disorders.

[7] Volkert J., Gablonski T.C., Rabung S. (2018). Prevalence of personality disorders in the general adult population in Western countries: Systematic review and meta-analysis. Br. J. Psychiatry.

[8] Beckwith H., Moran P.F., Reilly J. (2014). Personality disorder prevalence in psychiatric outpatients: A systematic literature review. Personal. Ment. Health.

[9] Hopwood C.J., Kotov R., Krueger R.F., Watson D., Widiger T.A., Althoff R.R., Ansell E.B., Bach B., Michael Bagby R., Blais M.A. (2018). The time has come for dimensional personality disorder diagnosis. Personal. Ment. Health.

[10] Grant B.F., Chou S.P., Goldstein R.B., Huang B., Stinson F.S., Saha T.D., Smith S.M., Dawson D.A., Pulay A.J., Pickering R.P. (2008). Prevalence, correlates, disability, and comorbidity of DSM-IV borderline personality disorder: Results from the Wave 2 National Epidemiologic Survey on Alcohol and Related Conditions. J. Clin. Psychiatry.

[11] Oldham J.M., Skodol A.E., Kellman H.D., Hyler S.E., Doidge N., Rosnick L., Gallaher P.E. (1995). Comorbidity of axis I and axis II disorders. Am. J. Psychiatry.

[12] World Health Organization (1978). International Classification of Diseases.

[13] Bach B., First M.B. (2018). Application of the ICD-11 classification of personality disorders. BMC Psychiatry.

[14] Tyrer P., Mulder R., Kim Y.R., Crawford M.J. (2019). The Development of the ICD-11 Classification of Personality Disorders: An Amalgam of Science, Pragmatism, and Politics. Annu. Rev. Clin. Psychol..

[15] Giourou E., Skokou M., Andrew S.P., Alexopoulou K., Gourzis P., Jelastopulu E. (2018). Complex posttraumatic stress disorder: The need to consolidate a distinct clinical syndrome or to reevaluate features of psychiatric disorders following interpersonal trauma?. World J. Psychiatry.

[16] Karatzias T., Bisson J., Roberts N., Shevlin M., Hyland P., Maercker A., Ben-Ezra M., Coventry P., Murphy P., Cloitre M. (2019). Psychological interventions for ICD-11 complex PTSD symptoms: Systematic review and meta-analysis. Psychol. Med..

[17] Bateman A., Tyrer P. (2004). Services for personality disorder: Organisation for inclusion. Adv. Psychiatr. Treat..

[18] Ferguson A. (2016). Borderline Personality Disorder and Access to Services: A Crucial Social Justice Issue. Aust. Soc. Work.

[19] Tyrer P., Reed G.M., Crawford M.J. (2015). Classification, assessment, prevalence, and effect of personality disorder. Lancet.

[20] Sheehan L., Nieweglowski K., Corrigan P. (2016). The Stigma of Personality Disorders. Curr. Psychiatry Rep..

[21] Watts J. (2019). Problems with the ICD-11 classification of personality disorder. Lancet Psychiatry.

[22] Gescher D.M., Kahl K.G., Hillemacher T., Frieling H., Kuhn J., Frodl T. (2018). Epigenetics in Personality Disorders: Today’s Insights. Front. Psychiatry.

[23] Amad A., Ramoz N., Thomas P., Jardri R., Gorwood P. (2014). Genetics of borderline personality disorder: Systematic review and proposal of an integrative model. Neurosci. Biobehav. Rev..

[24] Ruocco A.C., Carcone D. (2016). A Neurobiological Model of Borderline Personality Disorder: Systematic and Integrative Review. Harv. Rev. Psychiatry.

[25] De Genna N.M., Feske U., Larkby C., Angiolieri T., Gold M.A. (2012). Pregnancies, abortions, and births among women with and without borderline personality disorder. Womens Health Issues.

[26] Ferraro A.A., Rohde L.A., Polanczyk G.V., Argeu A., Miguel E.C., Grisi S., Fleitlich-Bilyk B. (2017). The specific and combined role of domestic violence and mental health disorders during pregnancy on new-born health. BMC Pregnancy Childbirth.

[27] Murphy C.C., Schei B., Myhr T.L., Du Mont J. (2001). Abuse: A risk factor for low birth weight? A systematic review and meta-analysis. CMAJ.

[28] Florange J.G., Herpertz S.C. (2019). Parenting in Patients with Borderline Personality Disorder, Sequelae for the Offspring and Approaches to Treatment and Prevention. Curr. Psychiatry Rep..

[29] Judd F., Komiti A., Sheehan P., Newman L., Castle D., Everall I. (2014). Adverse obstetric and neonatal outcomes in women with severe mental illness: To what extent can they be prevented?. Schizophr. Res..

[30] Guy N., Newton-Howes G., Ford H., Williman J., Foulds J. (2018). The prevalence of comorbid alcohol use disorder in the presence of personality disorder: Systematic review and explanatory modelling. Personal. Ment. Health.

[31] Nesari M., Olson J.K., Vandermeer B., Slater L., Olson D.M. (2018). Does a maternal history of abuse before pregnancy affect pregnancy outcomes? A systematic review with meta-analysis. BMC Pregnancy Childbirth.

[32] Newton-Howes G., Clark L.A., Chanen A. (2015). Personality disorder across the life course. Lancet.

[33] National Institute for Health and Care Excellence (2014). Antenatal and Postnatal Mental Health: Clinical Management and Service Guidance.

[34] Royal College of Psychiatrists (2015). Perinatal Mental Health Services. Recommendations for the Provision of Services for Childbearing Women.

[35] Staneva A., Bogossian F., Pritchard M., Wittkowski A. (2015). The effects of maternal depression, anxiety, and perceived stress during pregnancy on preterm birth: A systematic review. Women Birth.

[36] Lee H.C., Lin H.C. (2010). Maternal bipolar disorder increased low birthweight and preterm births: A nationwide population-based study. J. Affect. Disord..

[37] Mei-Dan E., Ray J.G., Vigod S.N. (2015). Perinatal outcomes among women with bipolar disorder: A population-based cohort study. Am. J. Obstet. Gynecol..

[38] Witt S.H., Streit F., Jungkunz M., Frank J., Awasthi S., Reinbold C.S., Treutlein J., Degenhardt F., Forstner A.J., Heilmann-Heimbach S. (2017). Genome-wide association study of borderline personality disorder reveals genetic overlap with bipolar disorder, major depression and schizophrenia. Transl. Psychiatry.

[39] Jablensky A.V., Morgan V., Zubrick S.R., Bower C., Yellachich L.A. (2005). Pregnancy, delivery, and neonatal complications in a population cohort of women with schizophrenia and major affective disorders. Am. J. Psychiatry.

[40] Howard L.M., Bekele D., Rowe M., Demilew J., Bewley S., Marteau T.M. (2013). Smoking cessation in pregnant women with mental disorders: A cohort and nested qualitative study. BJOG.

[41] Blankley G., Galbally M., Snellen M., Power J., Lewis A.J. (2015). Borderline Personality Disorder in the perinatal period: Early infant and maternal outcomes. Australas. Psychiatry.

[42] Chawanpaiboon S., Vogel J.P., Moller A.B., Lumbiganon P., Petzold M., Hogan D., Landoulsi S., Jampathong N., Kongwattanakul K., Laopaiboon M. (2019). Global, regional, and national estimates of levels of preterm birth in 2014: A systematic review and modelling analysis. Lancet Glob. Health.

[43] Quinn J.A., Munoz F.M., Gonik B., Frau L., Cutland C., Mallett-Moore T., Kissou A., Wittke F., Das M., Nunes T. (2016). Preterm birth: Case definition & guidelines for data collection, analysis, and presentation of immunisation safety data. Vaccine.

[44] Steer P. (2005). The epidemiology of preterm labour. BJOG.

[45] Raju T. (2017). The “Late Preterm” Birth-Ten Years Later. Pediatrics.

[46] Mangham L.J., Petrou S., Doyle L.W., Draper E.S., Marlow N. (2009). The cost of preterm birth throughout childhood in England and Wales. Pediatrics.

[47] Thorngren-Jerneck K., Herbst A. (2001). Low 5-minute Apgar score: A population-based register study of 1 million term births. Obstet. Gynecol..

[48] Cnattingius S., Norman M., Granath F., Petersson G., Stephansson O., Frisell T. (2017). Apgar score components at 5 minutes: Risks and prediction of neonatal mortality. Paediatr. Perinat. Epidemiol..

[49] Casey B.M., McIntire D.D., Leveno K.J. (2001). The continuing value of the Apgar score for the assessment of newborn infants. N. Engl. J. Med..

[50] Papile L.A. (2001). The Apgar score in the 21st century. N. Engl. J. Med..

[51] ACOG (2015). Committee Opinion No. 644: The Apgar Score. Obstet. Gynecol..

[52] Siddiqui A., Cuttini M., Wood R., Velebil P., Delnord M., Zile I., Barros H., Gissler M., Hindori-Mohangoo A.D., Blondel B. (2017). Can the Apgar score be used for international comparisons of newborn health?. Paediatr. Perinat. Epidemiol..

[53] Wardlaw T., Blanc A., Zupan J., Åhman E., UNICEF, WHO (2004). Low Birthweight: Country, Regional and Global Estimates.

[54] Hughes M.M., Black R.E., Katz J. (2017). 2500-g Low Birth Weight Cutoff: History and Implications for Future Research and Policy. Matern. Child. Health J..

[55] Risnes K.R., Vatten L.J., Baker J.L., Jameson K., Sovio U., Kajantie E., Osler M., Morley R., Jokela M., Painter R.C. (2011). Birthweight and mortality in adulthood: A systematic review and meta-analysis. Int. J. Epidemiol..

[56] Moher D., Shamseer L., Clarke M., Ghersi D., Liberati A., Petticrew M., Shekelle P., Stewart L.A., Group P.-P. (2015). Preferred reporting items for systematic review and meta-analysis protocols (PRISMA-P) 2015 statement. Syst. Rev..

[57] Nadelson S., Nadelson L.S. (2014). Evidence-based practice article reviews using CASP tools: A method for teaching EBP. Worldviews Evid. Based Nurs..

[58] Borjesson K., Ruppert S., Wager J., Bagedahl-Strindlund M. (2007). Personality disorder, psychiatric symptoms and experience of childbirth among childbearing women in Sweden. Midwifery.

[59] Pare-Miron V., Czuzoj-Shulman N., Oddy L., Spence A.R., Abenhaim H.A. (2016). Effect of Borderline Personality Disorder on Obstetrical and Neonatal Outcomes. Womens Health Issues.

[60] Baer R.J., Chambers C.D., Bandoli G., Jelliffe-Pawlowski L.L. (2016). Risk of preterm birth by subtype among Medi-Cal participants with mental illness. Am. J. Obstet. Gynecol..

[61] Kitai T., Komoto Y., Kakubari R., Konishi H., Tanaka E., Nakajima S., Muraji M., Ugaki H., Matsunaga H., Takemura M. (2014). A comparison of maternal and neonatal outcomes of pregnancy with mental disorders: Results of an analysis using propensity score-based weighting. Arch. Gynecol. Obstet..

[62] Skodol A.E. (2016). Personality pathology and population health. Lancet Psychiatry.

[63] Paris J., Black D.W. (2015). Borderline personality disorder and bipolar disorder: What is the difference and why does it matter?. J. Nerv. Ment. Dis..

[64] Ellard G.A., Johnstone F.D., Prescott R.J., Ji-Xian W., Jian-Hua M. (1996). Smoking during pregnancy: The dose dependence of birthweight deficits. Br. J. Obstet. Gynaecol..

[65] Kelly R., Russo J., Holt V., Danielsen B., Zatzick D., Walker E., Katon W. (2002). Psychiatric and substance use disorders as risk factors for low birth weight and preterm delivery. Obstet. Gynecol..

[66] Oni H., Khan M., Abdel-Latif M., Buultjens M., Islam M. (2019). Short-term health outcomes of newborn infants of substance using mothers in Australia and New Zealand: A systematic review. J. Obstet. Gynaecol. Res..

[67] Manuck T.A. (2017). Racial and ethnic differences in preterm birth: A complex, multifactorial problem. Semin. Perinatol..

[68] Blumenshine P., Egerter S., Barclay C.J., Cubbin C., Braveman P.A. (2010). Socioeconomic disparities in adverse birth outcomes: A systematic review. Am. J. Prev. Med..

[69] Malacova E., Regan A., Nassar N., Raynes-Greenow C., Leonard H., Srinivasjois R., Shand A.W., Lavin T., Pereira G. (2018). Risk of stillbirth, preterm delivery, and fetal growth restriction following exposure in a previous birth: Systematic review and meta-analysis. BJOG.

[70] Eke A.C., Saccone G., Berghella V. (2016). Selective serotonin reuptake inhibitor (SSRI) use during pregnancy and risk of preterm birth: A systematic review and meta-analysis. BJOG.

[71] Goldenberg R.L., Culhane J.F., Iams J.D., Romero R. (2008). Epidemiology and causes of preterm birth. Lancet.

[72] Stein D.J., Lund C., Nesse R.M. (2013). Classification systems in psychiatry, diagnosis and global mental health in the era of DSM-5 and ICD 11. Curr. Opin. Psychiatry.

[73] Tyrer P. (2014). A comparison of DSM and ICD classifications of mental disorder. Adv. Psychiatr. Treat..

[74] Morey L.C., Benson K.T. (2016). An investigation of adherence to diagnostic criteria, revisited: Clinical diagnosis of the DSM-IV/DSM-5 Section II personality disorders. J. Pers. Disord..

[75] Stepp S.D., Whalen D.J., Pilkonis P.A., Hipwell A.E., Levine M.D. (2012). Children of mothers with borderline personality disorder: Identifying parenting behaviors as potential targets for intervention. Pers. Disord..

[76] Cannon M., Jones P.B., Murray R.M. (2002). Obstetric complications and schizophrenia: Historical and meta-analytic review. Am. J. Psychiatry.

[77] Byrne M., Agerbo E., Ewald H., Eaton W.W., Mortensen P.B. (2003). Parental age and risk of schizophrenia: A case-control study. Arch. Gen. Psychiatry.

[78] Petfield L., Startup H., Droscher H., Cartwright-Hatton S. (2015). Parenting in mothers with borderline personality disorder and impact on child outcomes. Evid. Based Ment. Health.

[79] Grote N.K., Bridge J.A., Gavin A.R., Melville J.L., Iyengar S., Katon W.J. (2010). A meta-analysis of depression during pregnancy and the risk of preterm birth, low birth weight, and intrauterine growth restriction. Arch. Gen. Psychiatry.

[80] Rusner M., Berg M., Begley C. (2016). Bipolar disorder in pregnancy and childbirth: A systematic review of outcomes. BMC Pregnancy Childbirth.

[81] Mental Health Taskforce (2016). The Five Year Forward View for Mental Health: A Report from the Independent Mental Health Taskforce to the NHS in England.

